# Spatial statistics is a comprehensive tool for quantifying cell neighbor relationships and biological processes via tissue image analysis

**DOI:** 10.1016/j.crmeth.2022.100348

**Published:** 2022-11-21

**Authors:** Huw D. Summers, John W. Wills, Paul Rees

**Affiliations:** 1 Department of Biomedical Engineering, Swansea University, Swansea SA1 8QQ, UK; 2 Department of Veterinary Medicine, University of Cambridge, Cambridge CB3 0ES, UK

**Keywords:** cell imaging, spatial statistics, tissue analysis, cytometry

## Abstract

Automated microscopy and computational image analysis has transformed cell biology, providing quantitative, spatially resolved information on cells and their constituent molecules from the sub-micron to the whole-organ scale. Here we explore the application of spatial statistics to the cellular relationships within tissue microscopy data and discuss how spatial statistics offers cytometry a powerful yet underused mathematical tool set for which the required data are readily captured using standard protocols and microscopy equipment. We also highlight the often-overlooked need to carefully consider the structural heterogeneity of tissues in terms of the applicability of different statistical measures and their accuracy and demonstrate how spatial analyses offer a great deal more than just basic quantification of biological variance. Ultimately, we highlight how statistical modeling can help reveal the hierarchical spatial processes that connect the properties of individual cells to the establishment of biological function.

## Teaser

In this perspective, Summers et al. explore the application of spatial statistics to assess cellular relationships within microscopy images of biological tissue. They highlight the need for careful consideration of the multiscale structural heterogeneity of tissues and the need to be aware of these different scales when choosing a suitable test statistic.

## Introduction

Since the first demonstrations of the microscope by Robert Hooke and Antonie van Leeuwenhoek in the 17^th^ century,^1^ the analysis of cell images has been a mainstay of biological science. For most of the past few hundred years this analysis would have provided qualitative, descriptive information as the expert biologist deciphered the image in their microscope eyepiece and hand-recorded it on paper. Now, of course, digitization provides us with quantified images, stored as a matrix of pixel values, upon which we can perform unlimited mathematical manipulations and assessments. This technical advancement continues: in the past decade the application of machine learning algorithms has provided automated cell identification and feature extraction,^2^^,^^3^^,^^4^ while alternative imaging modalities can now display spatially resolved proteomics and genomics.^5^^,^^6^^,^^7^ Thus, today, accurate spatial data on cells, their neighbors, and their tissue environment are a mainstay of cell biology. The task of extracting information and knowledge from these measurement sets necessitates passing judgment on the reliability of the data and on their ability to differentiate the patterns of biological structure and process from random variations. This is where the calculation of spatial statistics becomes paramount in providing quantified confidence values for the variations seen within an image. Here we focus on the application of statistical techniques to tissue-image data. We address topics in spatial correlation, spatial mapping, and spatial structure and process, and highlight four important areas in the analysis of spatial data: (1) tissue classification, the statistical assessment of the tissue microenvironment; (2) spatial correlations, the assessment of the position dependence of multivariate cell factors; (3) neighbor and neighborhood relationships, the quantification of cell clustering; and (4) tissue morphology metrics, the quantification of the global patterns of biological mechanisms and processes.

## A brief, selective history

Much of the foundational work on spatial statistics was done in the 1950s through the 1970s, and its legacy is visible in the named metrics that are commonly used today. For example, we have P.A.P. Moran’s “I” index,^8^ a correlation metric between two cells, based on their respective distances from the population centroid; R.C. Geary’s “c” index,^9^ a correlation metric determined by the direct separation distance of two cells; and B.D. Ripley’s “K” index,^10^ a measure of cell density across increasing spatial ranges. Widespread application of these mathematical techniques to practical applications was enabled by the growing analytical capability of computers and focused on the spatial features of landscapes. In geography, statistical analysis provided urban planners with tools to manage building development and the associated traffic flows^11^; in economics, product supply and distribution networks could be optimized^12^; and in ecology, the spatial interactions of species within an environment could be quantified.^13^ The field of spatial statistics is therefore well established in the geographical sciences, with mathematical tools now widely available in comprehensive and well-established software packages referred to as geographical information systems (GIS).^14^^,^^15^ For example, spatstat provides simulations of point process models and parametric and non-parametric regression,^16^ and GeoDa provides local and global spatial correlation and cluster analysis.^17^

The quantitative analysis of image data for cell biology is, by comparison, a relatively recent phenomenon, with the first detailed reports appearing in the early 2000s^18^^,^^19^ and open-source software such as CellProfiler becoming available in 2006.^20^ However, progress over the past 20 years has been rapid, and automated cell segmentation is now routine in both cultured cells^21^ and tissue section images,^22^^,^^23^ facilitated by, for example, pixel-level classification using deep-learning artificial neural networks.^24^ Early efforts in this field concentrated on nucleus identification and then watershed cell outline segmentation using fluorescent molecules to additionally label specific sub-cellular targets.^25^ The current state of the art now allows multiplexed imaging in 40 or more channels, with advanced techniques such as imaging mass cytometry providing protein mapping^7^ and spatial transcriptomics enabling single-cell identification of gene expression.^5^ While single-cell imaging capabilities advance, the expanding use of digital pathology and the development of 3D *in vitro* cell cultures (e.g., organ on a chip) call for the analysis of cellular relations within the context of complex, heterogeneous tissue environments.^26^

The acquisition of dense multiplexed datasets from modern microscopy has triggered the development of a wide range of computational tools for image analysis, many of which provide spatial statistical measures. For example, within the widely used, open-source image processing platform ImageJ (https://imagej.nih.gov/ij/), there are plug-ins for point pattern analysis (Spatial statistics 2D/3D^27^), assessment of object interaction through spatial interaction potentials (MosaicIA^28^), and machine learning tools (deepImageJ^29^), to name but a few. A selection of other widely used open-source packages is provided in Table 1.

**Table 1:** A selection of open-source software packages for statistical analysis of cell images

Package	Capability	Year	Reference
Squidpy	spatial graphing, neighborhood proximity tests, spatial autocorrelation tests	2022	https://pypi.org/project/squidpy/^30^
Giotto	spatial correlation, spatial domain detection, pattern simulation	2021	https://rubd.github.io/Giotto_site/^31^
ImaCytE	interactive visual analysis of cell microenvironment	2021	https://github.com/biovault/ImaCytE^32^
Spark	spatial regression using generalized linear spatial models (GLSMs)	2020	https://xzhoulab.github.io/SPARK/^33^
SpatialDE	Gaussian process regression	2018	https://github.com/Teichlab/SpatialDE^34^
histoCAT	neighborhood analysis: permutation test of local interaction pairs compared with random distributions	2017	https://bodenmillergroup.github.io/histoCAT/^35^

For an extensive list of available software see https://htmlpreview.github.io/?https://github.com/drieslab/awesome-spatial-data-analysis/blob/main/review_spat_trns_methods.html.

It is clear that the development of spatial statistics for cell biology is well beyond its infancy; there is a multiplicity of analysis tools, providing local and global statistical indices that are well proven. However, the field has not yet reached the maturity of geographical analysis, where spatial statistics are commonplace, being part of the standard mathematical toolset of the discipline. We are some way from realizing single-cell measures that are as ubiquitous as the p value is in describing statistical relevance at the population level. While there will undoubtedly be further development of the mathematical tools for statistical assessment, in light of the substantial capability already available, we focus in the following sections on the challenges and opportunities relating to the *application* of statistical measures for cellular analysis. We consider which statistic is most appropriate for a given image, the use of local (cell-to-cell) and global (population) metrics, and the influence of tissue environment and look forward to possibilities for data-driven, statistics-based models of cell transport and behavior in tissue.

## Assessing cells within the heterogeneous tissue landscape

**Figure 1:**
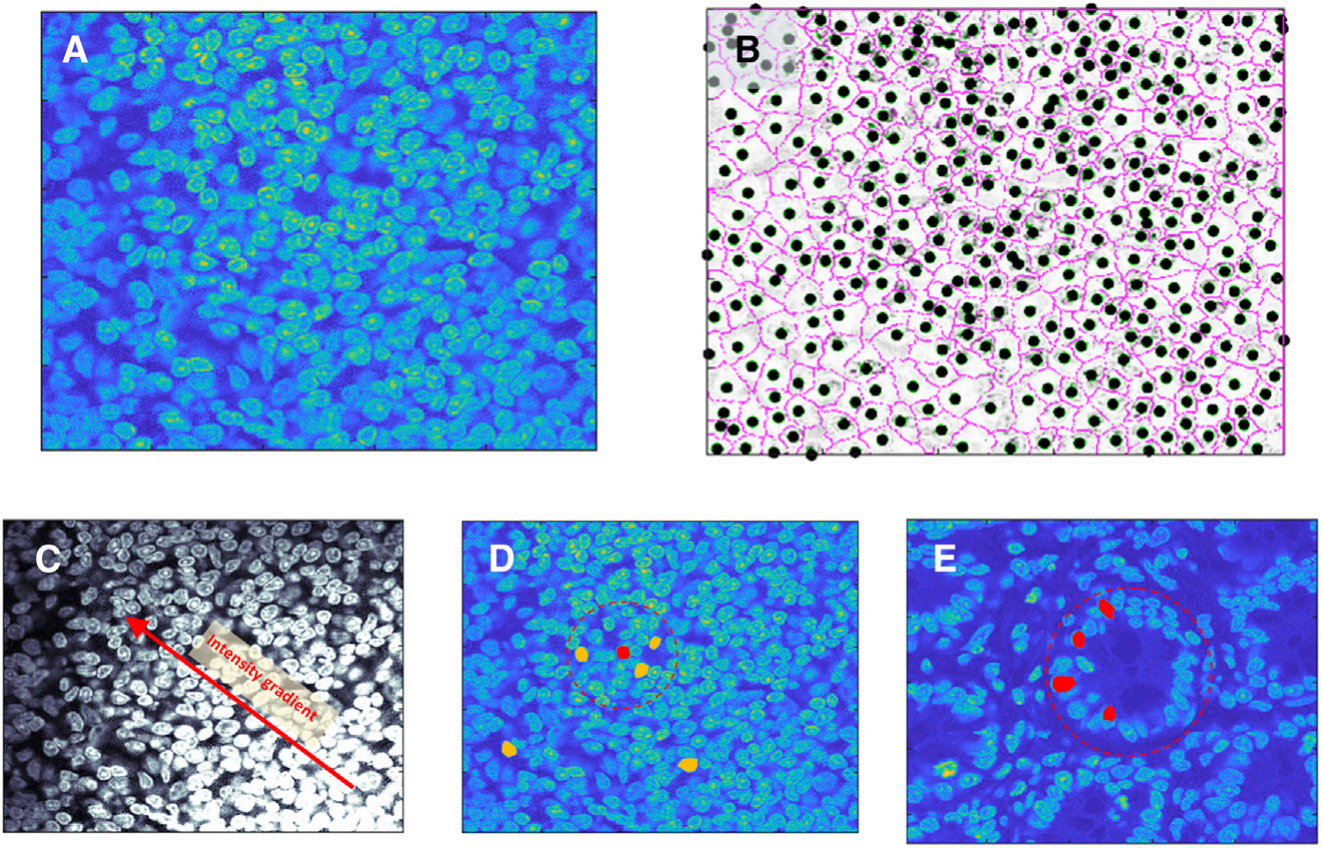
Considering local and global spatial statistics and the influence of the tissue environment (A and B) Computerized image analysis now readily provides segmentation of cell nucleus and cytoplasm in tissue and the location of the centroid position. In this example, (A) is a typical field of cells for which (B) shows the cell outlines (magenta line) and centroid positions (black circle) obtained by segmentation based on fluorescent staining of membrane and nucleus. (C and D) Within quasi-homogeneous tissue, statistical analysis can be applied in a straightforward manner to identify spatial correlations of cell phenotypes or spatial patterning in cell markers. For example, in (C), a spatial gradient in pixel intensity is shown, such as would result from differing expression of fluorescent molecular biomarkers; while (D) depicts the definition of a local neighborhood (red dashed circle) within which the numbers of cells of specific types (shown in yellow and red) may be assessed for comparison to a hypothesis of random distribution. (E) However, many biological samples will display a heterogeneous cellular environment, and in this case, care must be taken. Here, the image shows a tissue cross section with a clear meta-structure in which the cell positions are primarily determined by the tissue morphology. Now the definition of a cell neighborhood will lead to a test statistic for the selected cells (highlighted in red) that is highly significant, i.e., indicative of a pattern that is far from random. We need to be aware that, while we have a significant statistic, we do not have a significant result; this is just a reflection of the underlying tissue morphology rather than a directed biological response.

Spatial analysis of tissue begins with the identification of cell outlines (cell segmentation) and centroid locations (Figures 1A and 1B) and the extraction of shape, intensity, and position features. From these a range of statistical measures can be calculated at local and global levels (Figures 1C and 1D). However, if meaningful conclusions are to be drawn from the statistics, the heterogeneity of the tissue must be considered. Care is needed in this respect, for many spatial indices assume underlying homogeneity of the imaged area, considering the frequency of occurrence of cells within a specified tissue region and then comparing this with a null hypothesis of random distribution. For example, Ripley’s K statistic assesses whether the number of cells within an area of radius *r* is proportional to *πr*^*2*^, as expected for a spatial Poisson process.^10^ Thus, when assessing the question of statistical relevance, an expectation value (statistically most likely) for the test statistic is often calculated via random permutation of objects across all possible locations.^36^^,^^37^ However, for cells within tissue, the null hypothesis of randomization is not useful, as we may expect considerable heterogeneity (global spatial autocorrelation) as the norm (Figure 1E). Indeed, for tissues with heterogeneous structure, areas of the image may be inaccessible or unoccupied by cells (e.g., luminal regions), and this context must be corrected for in the analysis method.

Discussion of the issue of non-homogeneity of the sample space and its influence on measures of statistical significance can be found within the literature on geographical spatial analyses.^38^^,^^39^ There are means by which a heterogeneous environment can be statistically assessed. In particular, Monte Carlo techniques can be used to randomly allocate cell phenotype onto the positional grid obtained by cell segmentation,^31^^,^^40^ i.e., a randomization onto the measured spatial pattern. These random sets can then be used to define the null distribution of the test statistic, with which measured values may be compared using p values or similar indicators of significance. However, the challenges posed by spatial heterogeneity remain underappreciated and so care must be taken when interpreting spatial indices, else statistical significance may constitute a positive test of biological activity (e.g., immune cell congregation) or just refelct the underlying tissue morphology (e.g., high epithelial cell density within membranes). In Figure 1E, for instance, the chosen circular intestinal crypt region exhibits a marked clustering of cells for which colocation metrics would be high. However, this is merely a reflection of the tissue morphology and would not signify a biologically surprising congregation of cells. Alterations to the size of the defined “local” region in this example would also produce high variation in the test metric due to the underlying morphology.

All of the above highlights the need to recognize, quantify, and classify tissue morphology, so that justifiable comparisons can be made in the knowledge that a common tissue structure is being assessed. The ability to routinely segment cells within an image and to quantify cell position provides a powerful analysis tool, which can, and should, be implemented as part of basic microscopy assessment. A rudimentary demonstration of the benefit of this is discussed below with an example.

**Figure 2:**
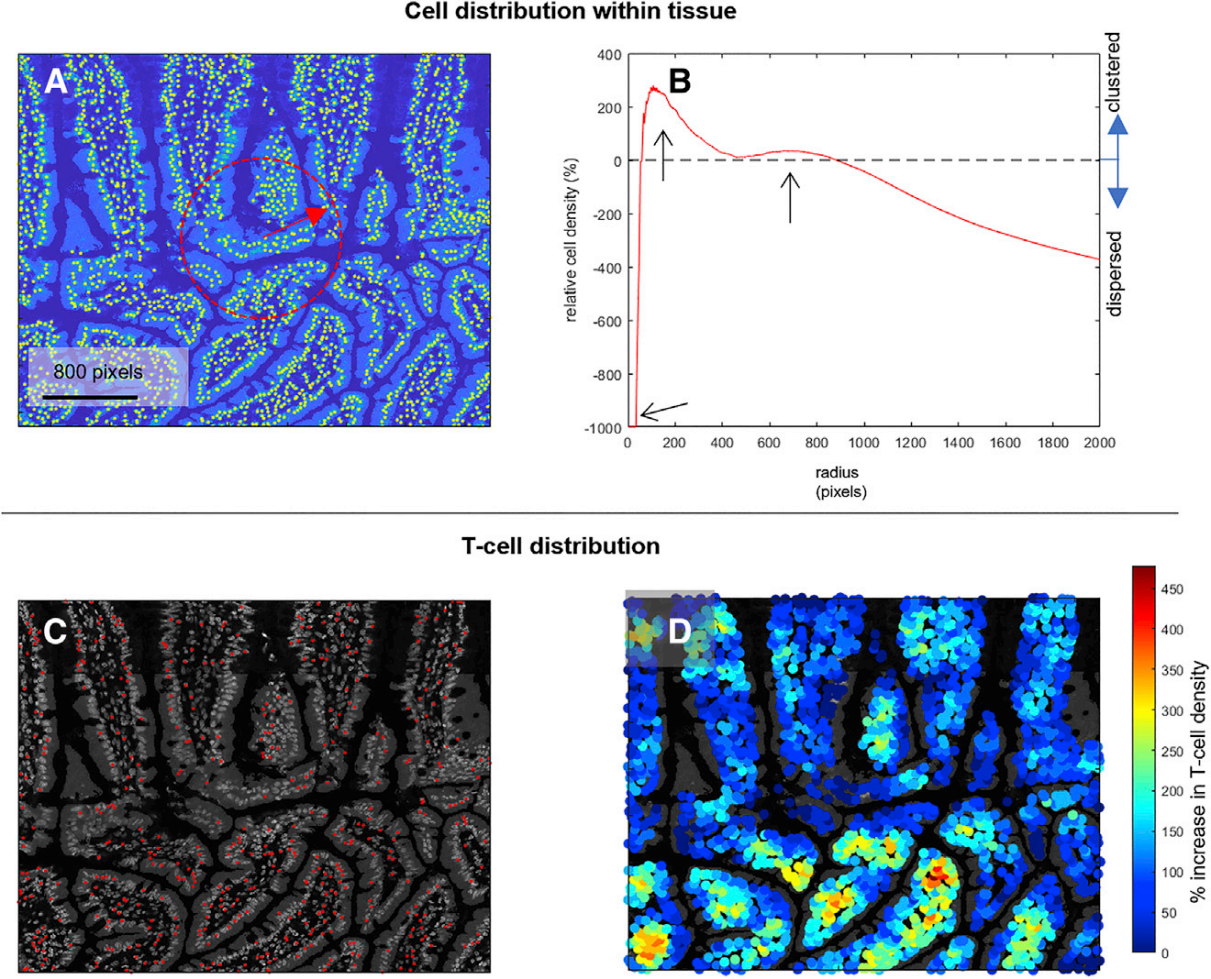
Extracting spatial metrics from tissue images (A) Highly informative statistics can be extracted from straightforward calculation of spatial cell density. The image shows a section of mouse ileum where the centroids of all cells are highlighted in yellow. These can be used to calculate cell density within a given region, in this case, the circular region indicated in red. (B) The result for image (A) is plotted in (B) as the relative percentage increase in area density compared with the random distribution value. Three features are immediately apparent in the plot (shown with arrows): (1) no cells within a radius of 30 pixels, as this is the minimum cell diameter; (2) a peak at ∼100 pixels corresponding to the typical cell separation within the large cell clusters; and (3) a second peak at ∼800 pixels corresponding to the mean separation of the clusters themselves, i.e., the spacing introduced by the presence of voids in the tissue. (C) The same image from (A) can be analyzed to show only a specific sub-set of cells (T cells, identified by the presence of the CD3 cell differentiation marker and with centroid positions highlighted in red). Here, any deviation from random spatial patterning cannot be reliably ascertained by eye. (D) When the image is re-plotted using a false color scale to display the local T cell density, this re-imaging clearly shows higher than expected T cell numbers within the lower half of the section.

Basic quantification of an image can provide immediate insight into the spatial distribution of cells. In the example shown in Figure 2, visual inspection of the image in which the centroids of all cells within a section of mouse ileum are highlighted in yellow (Figure 2A) shows a morphology that is typical of gut tissue, with clearly defined clusters of cells separated by voids. This inhomogeneity in cell distribution can be readily quantified by calculating the density of cells within a circular area of increasing radius, located at the center of the image (the core concept of Ripley’s K statistic). For a random spatial distribution, we would expect this metric to be constant and equal to the total cell number divided by total area. Spatial patterning introduces non-linearity to the cell number-area relationship and is identified by the presence of maxima in specific radial bands. The result for the image in Figure 2A is plotted as the relative percentage increase in area density compared with the random distribution value (Figure 2B), and three features become apparent from the plot: (1) no cells are within a radius of 30 pixels, as this is the minimum cell diameter and hence the minimum packing distance; (2) a peak at ∼100 pixels corresponds to the typical cell separation within the large cell clusters; and (3) a second peak at ∼800 pixels corresponds to the mean separation of the clusters themselves, i.e., the spacing introduced by the presence of voids in the tissue. The same image can be analyzed to show only a specific sub-set of cells, such as T cells (Figure 2C). Here, the spatial distribution of cells is not so easily assessed by eye; the T cells are present throughout the tissue section, but it is difficult to make a judgment on whether this is an even spread. Straightforward calculation of cell density can again provide valuable quantitative information. If the image is re-plotted, this time to show all cells, and a false color scale is used to display the local T cell density (T cells/area in a circle centered on each cell, with radius equal to 10× the mean cell diameter), this now clearly shows higher than expected T cell numbers within the lower half of the section (Figure 2D). This shows how simple cell density measurements can provide quantitative information that cannot be extracted from visual inspection alone.

**Figure 3:**
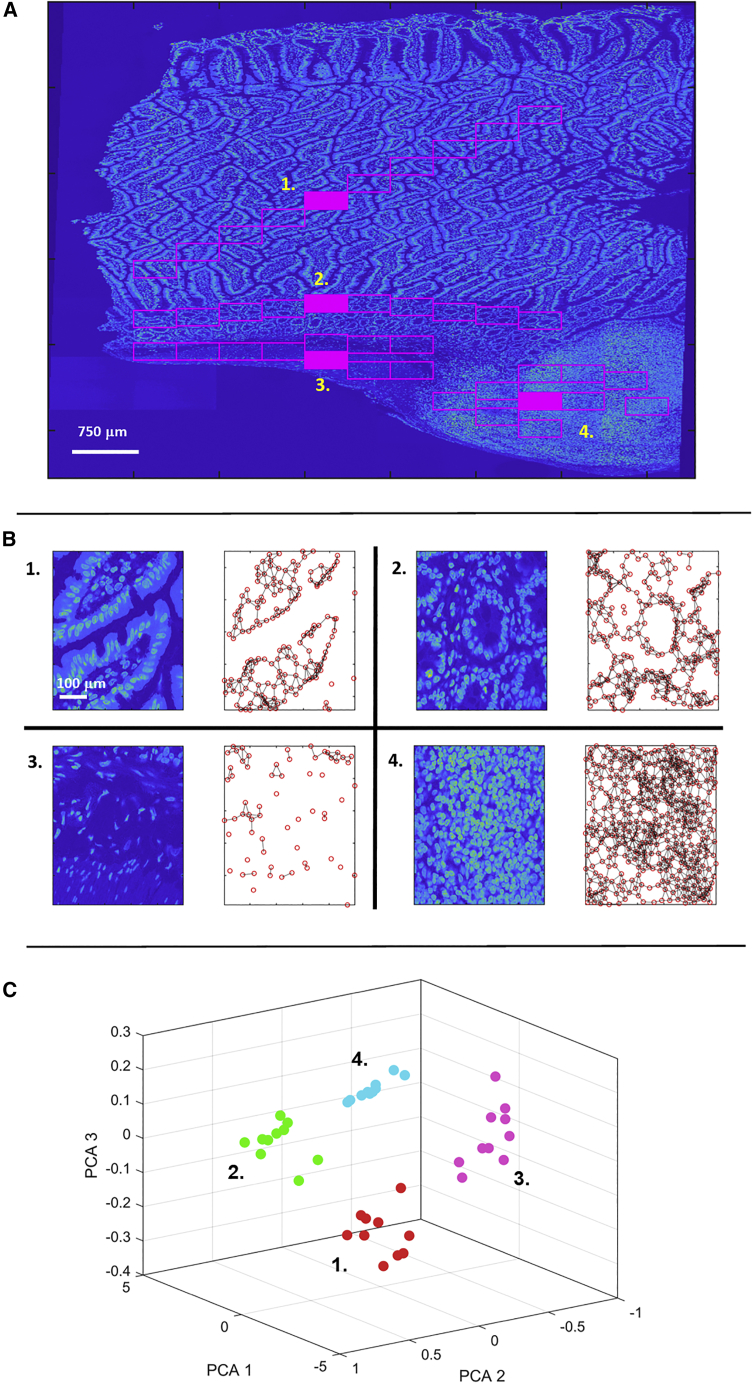
Using geometric graphs to classify and differentiate tissue (A) The image shows a longitudinal section of mouse ileum containing a Peyer’s patch lymphoid follicle. Four distinct regions, consisting of the villous mucosa, villous crypts, muscularis layer, and lymphoid tissue, are marked on the image (labeled 1 to 4). The magenta outlines define 10 sub-fields within the image, for each of the four tissue regions. (B) Left and right images show the transformation of image fields to graph networks with nodes defined by cell centroid positions and edges joining cells (representing the four tissue regions indicated by the filled magenta sections in [A]). (C) A principal-component analysis of the 40 image sub-sections (magenta sections in [A]), in which the different tissue regions clearly appear as differentiated clusters.

A more sophisticated analysis of spatial relationships can be gained by using geometric graphs to classify and differentiate tissue. As an example, we look at a longitudinal section of mouse ileum containing a Peyer’s patch lymphoid follicle (Figure 3). This gut tissue has a diverse morphology with different local structures visible for the villous mucosa (region 1), villous crypts (region 2), muscularis layer (region 3), and lymphoid tissue (region 4) (Figure 3A). Clearly, analysis of this image cannot proceed under the assumption of spatial homogeneity. One route to quantifying tissue geometry and therefore classifying local structure is to use graph theory,^41^ whereby transformation of cell centroid positions to a graph provides an abstracted and quantified description of the tissue (Figure 3B). Multivariate data matrices can be constructed using a range of graph metrics (e.g., number of nodes, fraction of end and isolated nodes, shortest path between nodes, node eccentricity, etc.), which describe both local and global cell-cell relationships. Applying data clustering and dimensional reduction techniques to the graph data matrix provides identification and visualization of similar tissue regions based on their multivariate graph metrics. In this example, we can see that a principal-component analysis of 40 areas from the sections depicted in the image of the mouse ileum shows the different tissue regions clearly as differentiated clusters (Figure 3C). Hence, here we see that the detailed image of the tissue becomes transformed to a simplified representation of points (nodes) at the cell centroid positions, joined by straight lines to nearest neighbors (edges). This translation of a segmented-cell image to a graph of nodes and edges provides a powerful and computationally efficient analysis tool, for it captures the important cell-to-cell relationships in a simple mathematical construct, whose various metrics provide a generalized numerical description of tissue morphology. Approaches such as this can identify regions of similar topology within the heterogeneous structure of a single tissue section or establish structural similarity in multiple images from different samples, e.g., across a patient study set. Again, open-source software is available to aid in the conversion of a tissue image to a node network, e.g., cytoNet.^42^

Ultimately, the important point to remember is that any statistical assessment of spatial pattern in tissues is inherently multiscale, influenced by local cell-to-cell interactions and the globally coordinated behavior of many cells. We therefore need to be aware of these different scales when choosing a suitable test statistic and in interpreting its value.^43^

## Spatial correlations

A substantial part of the literature on spatial analysis in cell biology describes the application of test statistics at a population level.^33^ Here, the analysis of spatial relationships is not resolved in a position-dependent manner; rather, valuable biological insight is gained through a more generalized identification of those cell-to-cell interactions that depend upon spatial location. In these approaches, the correlation of position to other cell metrics is assessed using mixed model regression techniques with the aim being to establish whether there is a general spatial dependence of the observed characteristic rather than trying to spatially resolve the biology.^44^ The general approach when using regression techniques is to assume that observations are independent, but if there is a hidden spatial dependence between the correlates, then this no longer holds, and the regression residuals will not be random, i.e., they will contain a spatial weighting. For example, comparison of immune cell phenotypic markers is highly likely to produce distance-dependent regression residuals, as the need for molecular transport between cells necessitates a spatial aspect to immune response.^45^

While mathematically spatial regression can be implemented in various forms, the general hypothesis underlying the technique can be understood through the concept of a spatial lag.^46^ This is represented by a term, *q*_*i,j*_ = ρ*w*_*i*_*y*_*j*_, which captures the relationship of the dependent variable, *y*, on its value at nearby locations (indexed by *j*). Here, *w*_*i*_ is a spatial weights matrix and ρ a spatial coefficient, determining the strength of the spatial effect. The index, *i/j*, refers to specific cells. By including *q*_*i,j*_ in the regression equation, the distance of a cell to its neighbor becomes a predictor variable rather than a residual effect:

(Equation 1) $$y_{i}=x_{i}\beta +\sum_{j}q_{i,j}+u_{i}.$$

The *x*_*i*_*β* term captures the spatially independent component of the correlation of *y*_*i*_ with the explanatory variable, *x*_*i*_, and *u*_*i*_ now represents a true random distribution of the regression residuals, i.e., the fundamental random variability in the correlation variables.

Regression analysis has been heavily used in spatial transcriptomics to assess positional dependencies in gene expression.^31^^,^^47^ Here a variety of Gaussian process models have been used to quantify gene expression covariance through pairwise distance measures of spatial points.^34^ The specific mathematical form chosen for the spatial variance term, *q*_*i,j*_, captures the behavior of the underlying spatial processes. Thus, this approach can provide biological insight as well as data statistics, with the correspondence of the measurement set to different variance functions being assessed, i.e., do cell-to-cell correlations follow an exponentially decaying, linear or periodic spatial dependency? Examples of applications could include the study of metabolic zoning in the liver, analysis of cell signaling gradients, or macrophage translocation of ingested particles. Following work in geostatistics,^48^ generalized linear spatial models (GLSMs) have also been used for regression analysis of non-Gaussian data.^33^ This removes assumptions as to the underlying structure of the data and so can provide more robust hypothesis testing in situations of low sample/count number.

## Neighbor and neighborhood relationships

Perhaps the most compelling application of spatial statistics in tissue imaging is in the generation of spatially resolved metrics, enabling the mapping of statistical variations across the tissue. These local statistics of cell association provide a visualization of the cell neighborhood that is so important in determining the biology of the tissue microenvironment, e.g., identifying cell clustering or spatial patterning due to signaling gradients. The basic spatial concept in constructing local metrics is the cell neighborhood: an area *a*, centered on cell *i*, the cell of interest, and defined by a radius, *r*. The construction of a test statistic involves calculation of distance-dependent indices, relating cell *i* to other chosen cells within area, *a*. These may be all other cells in the neighborhood, e.g., in measures of cell clustering, or involve specific sub-sets of cells, e.g., in assessing colocation of different phenotypes.

To understand the general mathematical approach to statistical testing of local spatial correlation, we present a generalized form of metric that captures the basic principles:

(Equation 2) $$\text{Γ}=\sum_{i,j}C_{ij}W_{ij}.$$

The cell-cell relationships within the locality are quantified by *C*_*ij*_, a matrix containing the values of the chosen association metric, *x*, between pairs of cells, identified by the indices *i* and *j*. The particular form of the association metric can vary, and so *C*_*ij*_ can be populated in a variety of ways, depending upon the manner in which spatial interactions are quantified. For example, for Moran’s *I*, it is a product of cell distance from a mean population center, $C_{ij}=\left(x_{i}-\bar{x}\right)\left(x_{j}-\bar{x}\right)$, whereas for Geary’s c it is determined by the direct separation of cells *i* and *j*, $C_{ij}={\left(x_{i}-x_{j}\right)}^{2}$. The summation sign in Equation 2 tells us that the association metric is calculated for all cells, *i* and *j*, i.e., it calculates the relationship of every cell to all others in the image field. To invoke locality, therefore, a spatial filter is required. This is provided by *W*_*ij*_, a weight matrix describing the relationship between points *i* and *j*. *W*_*ij*_ acts as a selection filter because its value at specific *i* and *j* indices determines the scale of the correlation. For example, a binary *W*_*ij*_ will have matrix values equal to 1 for the *j* points that lie within the local region, *a*, of point *i*, and equal to 0 for all points outside of *a*. This therefore limits calculation of the test statistic for cell *i*, to consider only those relationships occurring within the defined local area. Other forms of *W*_*ij*_ can provide alternative filter functions, e.g., a distance-dependent exponential decay in association strength between cells *i* and *j* provides a statistic based on all relationships but with declining influence of the more distant cells. The metric, Γ, in Equation 2 quantifies the degree of spatial association between cells, but it provides us with no information on whether this association is statistically significant, i.e., there is no comparison provided through which we can assess if the measured association is unexpected. In the full equation for a spatial statistic, therefore, expressions such as Γ become the numerator in a larger expression, in which the denominator determines the scale, and therefore the relevance, of any spatial association.

**Figure 4:**
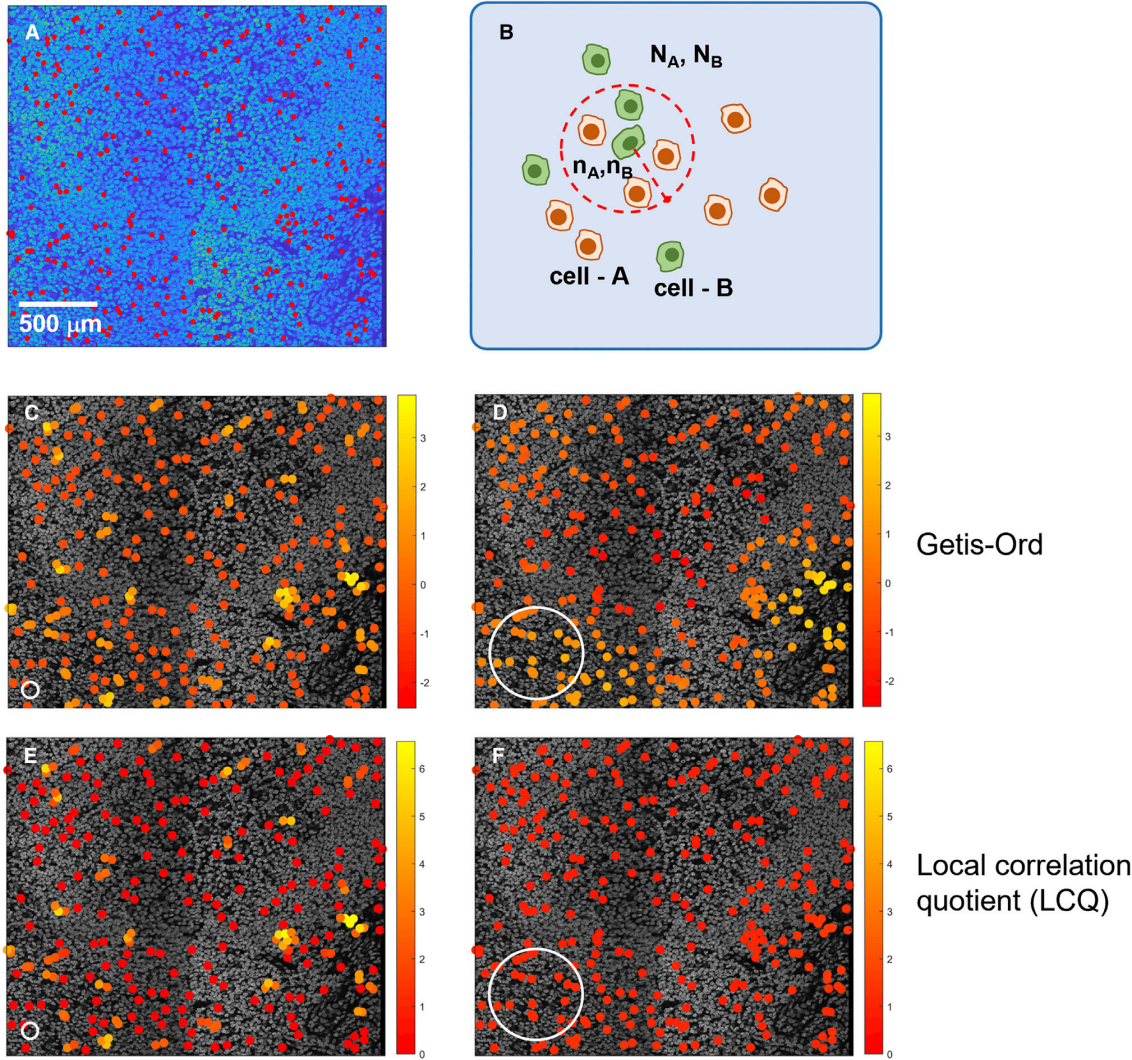
The statistics of the cell neighborhood (A) A hypothetical sub-population of cells is generated by randomly selecting ∼100 cells from a segmented image of a lymphoid tissue (identified by red circles). Analysis can then be undertaken to assess the spatial distribution of the 100 chosen cells (referenced as cell - B) compared with the whole of the cell population (referenced as cell - A). (B) Local neighborhood statistics are calculated by defining a circular region around each cell of type B and counting the number of cells of each type within this region (n_A_ and n_B_) and the total number in the image (N_A_ and N_B_). (C–F) The Getis-Ord z index (C and D) and the local colocation quotient (LCQ) (E and F) are calculated for different scales of local neighborhood (size indicated by a white circle in the lower left of [C]–[F]) and used to assess whether there is clustering of a particular cell type. The cell - B sub-population is false colored according to the values of the relevant spatial statistic. The resulting images are highly dependent upon the metric chosen and the length scale used in its calculation.

Local spatial statistics can be disarmingly simple to implement and convey immediate visual impact; however, they can also be subtle and complex in their formulation, and care must be taken when interpreting them. An example highlighting this point is presented in Figure 4, which compares two metrics, the Getis-Ord statistic (GO)^49^ and the local colocation quotient (LCQ).^50^ For a hypothetical population of cells generated by randomly selecting ∼100 cells from a segmented image of a lymphoid tissue (Figure 4A), both GO and LCQ are calculated (Figure 4B) for different scales of local neighborhood (Figures 4C–4E). Both metrics follow the common metrological approach of local statistics in counting cell frequency within the neighborhood of each cell of the chosen phenotype, in this case, the occurrence of cell type A within a circular region of radius *r* around each of cell type B. While the two metrics share a common philosophy, their mathematical forms are quite different:

(Equation 3) GO=nBNB−nANAσ(nBNB),

(Equation 4) LCQ=nBnA(NB−1)(NA−1),

where N_A_ is the global number of all cells and N_B_ is the global number of cells of type B. n_A_ is the local number of all cells and n_B_ the local number of cells of type B.

The GO metric determines the degree of clustering by the difference between the fraction of cell type B and the fraction of all cells within the local region. By adopting a difference measure, the GO technique has the additional ability to show cell sparsity (n_B_/N_B_ < n_A_/N_A_) as well as clustering (n_B_/N_B_ > n_A_/N_A_). Relevance is indicated by comparison to the standard deviation in the local cell fraction across all measurement locations. The LCQ, on the other hand, has a numerator that calculates the proportion of neighborhood cells that are cell type B and a denominator that determines measurement relevance by comparison with the global proportion of cell type B. As the radius defining a cell’s local area is adjusted, the way in which the changing numbers of neighbor cells alter the statistics depends on the test metric chosen. Here, in the example shown in Figure 4, for the smaller neighborhood, the two metrics show similar maps with only a few cells showing significant clustering (Figures 4C and 4E), as expected given the random selection process. However, for larger local regions, the two statistics diverge (Figures 4D and 4F). The GO index continues to show some variation in local cell density, albeit at lower significance levels. The reduction in the metric numerator as the larger local region becomes more representative is offset by reduction also of the denominator, as variability across the larger localities is smaller. In contrast, the LCQ tends to unity as the larger locality radius promotes sampling homogeneity and the local populations are near identical to the global cell mix.

Moreover, area effects also come into play for cells close to the image edge, where incomplete coverage of the local neighborhood must be corrected for.^51^ To summarize, the various spatial statistics may look similar in their function but can provide quite different outputs dependent upon the specifics of their mathematical structure and implementation. Thus, the user must be aware when assessing the scientific import of spatial relationships and using them to draw conclusions about biological significance.

## Tissue morphology metrics

The linkage between biological function and spatial organization is well appreciated,^52^^,^^53^ and this provides an opportunity to move spatial statistics beyond quantifying numerical variance to using the mathematics to determine and predict biological processes by modeling. When employed at the tissue level, spatial statistics can capture the design principles and operational processes of the complex biosystem, elucidating cell differentiation in morphogenesis, for example,^54^ or quantifying cell phenotype interactions in disease progression.^55^ Many tissue characteristics stem from emergent properties of the system, and so a complete understanding requires a holistic view across the whole topology of the tissue. For this we need to shift from local cell-cell or cell-neighborhood statistics and consider the global relationships between each and every cell. The challenge is to build spatial interactions, and the relationships they describe, into the statistical framework, so that processes such as diffusion, or directed transport between source and sink, can be enumerated in terms of cell position. Once again, the geographical sciences provide a large literature of previous work of use to the cell biologist. Of particular interest are spatial interaction models, which describe movement and exchange between spatial locations and so quantify the outcomes of the system dynamics.^56^^,^^57^ In general terms, these may be seen as descriptions of the movement of populations and of information, processes that translate immediately into the biological sphere in considering cell migration and cell signaling. In these models the key conceptual foundation is of a spatial interaction between two points, *i* and *j*, that is inversely proportional to a distance vector, *d*^*β*^_*i,j*_.^58^ This provides a mathematical construct that is both straightforward to interpret and a powerful tool for spatial process modeling. The use of a distance-based vector immediately relates the strength of interaction to spatial proximity, while the exponent, *β*, encodes specific process forms. For example, *β* = 2 corresponds to “gravity models” in which a physics-based concept of interaction fields is used to describe spatial interactions that decline as 1/*d*^2^_*i,j*_ in analogy to gravitational field strength. Direction within an interaction is determined by the sign of *β*, where a positive exponent describes repulsive interaction forces leading to spatial dispersion, while a negative *β* describes attractive forces and spatial clustering.

Another set of mathematical tools that has been widely used for biological studies are point-process models,^59^ which have been deployed from the tissue level down to sub-cellular scales. They have strong compatibility with quantitative tissue studies as they combine the analysis of spatial process with statistical probability. The null hypothesis here is an assumption of a process that leads to random distributions of objects across space, i.e., spatial independence of the measurement set. This corresponds to a Poisson spatial process. Ripley’s K index quantifies the deviation of a set of points from such a random distribution, and an assessment of cell position was included in Ripley’s original paper.^10^ The Poisson distribution of cells in 3D has also been studied,^60^ as has the spatial distribution of cell mitosis.^61^ Extensions of this approach to describe more complex situations involve the inclusion of spatial dependence into the test statistic. For example, a common approach in adapting the point process to address position-dependent datasets is to use Cox processes. These are described by an altered Poisson statistic in which the intensity, *λ* (mean number of events per unit area), is not constant but becomes a spatially dependent variable. This describes a physical situation in which measurement variability is greater than expected for a random set, and the resulting probability prediction functions are known as overdispersed Poisson or negative binomial distributions.^62^ Examples of biological applications of these statistics include parasite counts in epidemiology^63^ and endosomal uptake of particles by mammalian cells.^64^^,^^65^ The point-process framework is highly adaptable, allowing tissue-structure descriptions that go beyond simple single-population cell distributions. For example, marked point-process models incorporate additional information linked to each spatial point; this allows discrimination between cell phenotypes so that their interactions in the context of different tissue structures may be investigated.^66^ The examples discussed throughout this perspective highlight an important aspect of tissue structure: much of the image in each case (Figures 1E and 3) does not contain cells. Point-process models are able to calculate the probability of absent points and so can account for voids caused, for instance, by stroma, necrotic tissue, or blood vessels intersecting the image plane.^67^

Another powerful process-based analysis models spatial interaction using Gibbs processes. Like Poisson and Cox processes, the analysis is based on probabilistic determination of object distributions, and is based on a conceptual framework from thermodynamics in which the existence of the system is in one of multiple possible states (i.e., occupancy of specific spatial positions) and is described by a probability density function, *P*. For spatial interactions between objects separated by a distance, *d*, we have the general formulation of^68^:

(Equation 5) P(d)=q(d)exp[−∅(d)],

where Ø is an interaction potential (described by a mathematical function) determining the strength of interaction and *q* is a density parameter describing the number of possible interactions at distance *d*.

To provide a concrete example, consider the interaction between two cell phenotypes, A and B. Here, the term exp[−Ø(*d*)] would describe the probability of interaction between a pair of disparate cells separated by a distance, *d*. The overall probability of interaction will also depend on the relative frequency of cell types A and B, and this is described by *q*(*d*). Thus, the overall probability expression combines the likelihood of a type A and a type B cell interacting across a distance *d* and the likelihood that a given pair of cells will be of disparate phenotypes. The particular form of Ø can be chosen from a range of options according to the scale, strength, and distance dependence of the process to be modeled. As for the spatial interaction models discussed earlier in this section, the sign of the interaction potential determines the direction of the spatial process. If Ø = 0, the interaction leads to a randomized Poisson distribution, while Ø > 0 produces spatial dispersion and Ø < 0 spatial clustering.

Statistics is most commonly used to discriminate or compare datasets, for example, to test a set of measurements against a null hypothesis or to evaluate local cell density. In the context of tissues, however, the examples above show that statistical metrics may be applied to integrate information so as to gain a holistic view. Many aspects of tissue function involve emergent properties understandable only through study of the coordinated action of all constituent cells. This synchronized behavior can be captured by statistical process-oriented models. Spatial statistics can, therefore, provide a comprehensive tool for systems biology, capable of quantifying the interaction of system components (e.g., molecules and cells) *and* the behavior of the whole system (the interactome of the whole tissue).

## Concluding thoughts

We have entered an age in which microscopy information is almost always captured digitally, and so any reporting of tissue image data should contain some element of statistical analysis. If cell-to-cell interactions are of interest, then their spatial statistics clearly need to be collected; however, even if single cells are the objects of study, wider spatial dependencies in the expression of cell markers need to be captured to unravel the complexities of the biological system. Much of present practice and scientific literature on spatial statistics comes from expert groups in response to the demands of specific techniques, e.g., the need to assess 40 parallel channels of data collected with sub-cellular resolution over many thousands of microns by an imaging mass cytometer. Routine spatial analysis for microscopy by the wider biology community is not yet established, and there is still a tendency to extract signal information from cells using segmentation but then to lose all morphological context of the cells to their environs as population-based cytometry approaches are implemented. However, the tools and resources for the task are available and are being further developed, and so our call to the community is to adopt, adapt, and integrate these statistical techniques into your analysis workflows. We finish with two quotes, chosen to broaden appreciation of the power of spatial statistics, and to encourage a view of them as powerful descriptors of process, not just quantifiers of current biological state recorded by an image:

… form ever follows function, and this is the law. Where function does not change, form does not change.—Sullivan^69^

Structure without function is a corpse; function without structure is a ghost.—Vogel and Wainwright^70^

These are salient reminders that pattern in biology always serves a purpose, and that the complex, multiscale processes and interactions that characterize biological organisms are evident in the blueprint of their tissue and cell morphologies—the morphologies that are quantitatively captured by spatial statistics.
